# Pressure Load: The Main Factor for Altered Gene Expression in Right Ventricular Hypertrophy in Chronic Hypoxic Rats

**DOI:** 10.1371/journal.pone.0015859

**Published:** 2011-01-05

**Authors:** Jonas D. Baandrup, Lars H. Markvardsen, Christian D. Peters, Uffe K. Schou, Jens L. Jensen, Nils E. Magnusson, Torben F. Ørntoft, Mogens Kruhøffer, Ulf Simonsen

**Affiliations:** 1 Department of Pharmacology, Aarhus University, Aarhus, Denmark; 2 The Water and Salt Research Center, Department of Anatomy, Aarhus University, Aarhus, Denmark; 3 Department of Theoretical Statistics, Institute of Mathematics, Aarhus University, Aarhus, Denmark; 4 Molecular Diagnostic Laboratory, Department of Clinical Biochemistry, Aarhus University Hospital, Aarhus, Denmark; Istituto Dermopatico dell'Immacolata, Italy

## Abstract

**Background:**

The present study investigated whether changes in gene expression in the right ventricle following pulmonary hypertension can be attributed to hypoxia or pressure loading.

**Methodology/Principal Findings:**

To distinguish hypoxia from pressure-induced alterations, a group of rats underwent banding of the pulmonary trunk (PTB), sham operation, or the rats were exposed to normoxia or chronic, hypobaric hypoxia. Pressure measurements were performed and the right ventricle was analyzed by Affymetrix GeneChip, and selected genes were confirmed by quantitative PCR and immunoblotting. Right ventricular systolic blood pressure and right ventricle to body weight ratio were elevated in the PTB and the hypoxic rats. Expression of the same 172 genes was altered in the chronic hypoxic and PTB rats. Thus, gene expression of enzymes participating in fatty acid oxidation and the glycerol channel were downregulated. mRNA expression of aquaporin 7 was downregulated, but this was not the case for the protein expression. In contrast, monoamine oxidase A and tissue transglutaminase were upregulated both at gene and protein levels. 11 genes (e.g. insulin-like growth factor binding protein) were upregulated in the PTB experiment and downregulated in the hypoxic experiment, and 3 genes (e.g. c-kit tyrosine kinase) were downregulated in the PTB and upregulated in the hypoxic experiment.

**Conclusion/Significance:**

Pressure load of the right ventricle induces a marked shift in the gene expression, which in case of the metabolic genes appears compensated at the protein level, while both expression of genes and proteins of importance for myocardial function and remodelling are altered by the increased pressure load of the right ventricle. These findings imply that treatment of pulmonary hypertension should also aim at reducing right ventricular pressure.

## Introduction

Pulmonary arterial hypertension is a heterogeneous group of disorders characterized by increased pulmonary artery pressure and resistance as a result of pulmonary vascular remodeling, active vasoconstriction, and in-situ thrombosis. The increased pressure load results in right ventricular hypertrophy representing an initial stage which can progress to failure and death [1]. The current treatments with prostaglandin (PGI_2_) analogues (e.g. epoprostenol), endothelin receptor antagonists (e.g. bosentan), and phosphodiesterase 5 inhibitors (e.g. sildenafil) have markedly improved the prognosis of the disease [2]. Direct inotropic effects of epoprostenol on the right ventricle was described [3], [4] and that bosentan reduces cardiac fibrosis and hypertrophy [5]. However, these treatments are mainly vasodilatory and antiproliferative, and hence targeting the vasculature rather than the right ventricle in pulmonary hypertension.

Parameters of right ventricular function such as cardiac index and mean right atrial pressure are the most important determinants of survival in pulmonary arterial hypertension [1]. Multiple signal transduction pathways are known to be involved in the remodeling of the heart [6]. Although it is a matter of debate which mechanisms are important for transition to failure and hypertrophy, failure of the left ventricle are characterized to varying degrees by changes in extracellular matrix composition, energy metabolism, contraction, adrenergic signaling, and calcium handling [7]. In the right ventricle from chronic hypoxic rats gene expression studies have suggested a switch of metabolic genes suggesting that the hypertrophic right ventricle changes from fatty acid to glucose oxidation [8], and a recent microarray study of the right ventricle from rats with monocrotaline-induced pulmonary hypertension suggested that pro-apoptotic pathways and intracellular calcium handling enzymes play a role for development of failure [9]–[11] while growth genes such as mitogen activated protein kinase (MAPK) are pivotal in compensated hypertrophy [9]. However, in contrast to the thick-walled left ventricle, the right ventricle has a concave thin wall opposite to the convex interventricular septum, and the anatomic response to pressure overload of the right ventricle is different from the left ventricle [1], hence suggesting that other signaling pathways may play a role for development of right ventricular hypertrophy in response to pressure load.

Global gene analysis has been employed to map the expression profile of cardiac hypertrophy in man [12] and in the lungs and peripheral blood cells from patients with severe pulmonary arterial hypertension [13], [14] as well as in lungs of mice with hypoxic pulmonary hypertension [15]. These types of global gene analyses are believed to be of significant value both for understanding and predicting disease processes also in pulmonary hypertension [16].

The present study investigated the changes in global gene expression by gene chip analysis during the development of right ventricular hypertrophy induced by chronic hypoxic pulmonary hypertension in rats. Most of the regulated genes in the hypoxic model were expected to be associated to the adaptive response to sustain right ventricular output, but some may be exclusively associated to hypoxia. Therefore, gene expression changes were also analyzed in rats undergoing pulmonary trunk banding (PTB), another animal model for pressure loading of the right ventricle. The alterations in expression of a subset of genes were confirmed by quantitative realtime polymerase chain reaction (qPCR), immunoblotting, and immunohistochemistry.

## Methods

### Ethics Statement

All animal procedures followed the revised NIH publication no. 86–23, entitled: “Principle of laboratory animal care”, and were performed according to the Danish legislation with permission (no. 2006/561–1160) from the Animal Surveillance Committee, The Danish Ministery of Justice.

### Animal models of right ventricular hypertrophy

48 male Wistar rats (10 weeks, weight approximately 290 grams) were divided into two groups of 24 animals. Each group was divided into subgroups of six animals. Four subgroups were maintained at hypobaric, hypoxic pressure. Four subgroups were maintained at normobaric, normoxic pressure and served as controls (Table 1). The hypoxic group was placed in a hypobaric chamber, where the ambient pressure was continuously held at 500 mbar which was equivalent to rats breathing 10% oxygen at standard pressure. The temperature in the chamber was maintained at 21–22°C and the chamber was ventilated with air at approximately 45 l min^−1^ through an inlet valve with the aid of a vacuum pump. The four subgroups were maintained in these hypoxic, hypobaric conditions for 1, 2, 3 or 4 weeks and studied immediately after removal from the chamber. The hypoxic chamber was opened once a week for approximately 30–40 minutes for cleaning and supplying purposes. Age-matched controls were maintained in similar but normoxic, normobaric chambers. All animals were provided with chow and water ad libitum.

**Table 1:** Experimental design.

Animal groups	Week 1	Week 2	Week 3	Week 4	Total	GeneChip
Intervention (hypoxia)	6	6	6	6	24	16*
Control (normoxia)	6	6	6	6	24	12*

	Week 2	Week 3	Week 5	Week 6	Total	GeneChip
Intervention (PTB)	5	6	6	6	23	4*
Control (Sham)	5	6	6	6	23	4*

*Three animals from each control and four animals from each hypoxic subgroup were used for GeneChip analysis. Four animals from each group either pulmonary trunk banded (PTB) or sham at week 5 were used for GeneChip analysis.

For the PTB experiment, 46 male Wistar rats were divided into two groups. One group underwent PTB operation, and the other group underwent sham operation. Sham operated animals underwent the same procedure as PTB rats except for banding of the pulmonary trunk. Animals were subdivided into 4 groups and examined 2, 3, 5 and 6 weeks after the operation. 4 Sham and 4 PTB animals were randomly selected for gene chip analysis (Table 1). The animal model for pressure loading of the right ventricle using PTB has been described in detail in a previous publication [17].

In the experiment with chronic hypoxic rats, body weight (BW) and systemic systolic blood pressure (SSBP) of the animals were measured on the day of sacrifice. SSBP was measured using the tail cuff method with a plethysmograph (Digital Pressure Meter LE 5000 with tail cuff). Before measurements, rats were preheated for 20 minutes at 35°C in their cages. Rats were then placed in a heated Plexiglas tube (35°C). In each rat, three subsequent measurements within a difference of 5 mmHg were made, and the mean values were used. For measurement of right ventricular systolic pressure (RVSBP), rats were fully anaesthetized with an intraperitoneal injection of Midazolam 0.825 ml/kg, Fentanyl 0.825 ml/kg and sterilized water 0.825 ml/kg. For maintenance, intraperitoneal injection of 100 µL Fentanyl was given every 30 minutes. The external jugular vein was isolated by blunt dissection and ligated (using Seralon® 3/0 – Serag Wiessner). A small hole was cut in the vein and through this a catheter (30 cm. Tygon micropore, OD: 0.76 mm, ID: 0.25 mm, Norton performance plastics, OH, USA) slightly bended in one end, was inserted and led into the right ventricle through the right atrium (marks on the catheter at four to five cm indicates this location). The pressure profile was simultaneously registered via a pressure transducer (MLT0699 Disposable BP Transducer, ADInstruments, CO, USA), an amplifier (ML118G Quad Bridge Amp, ADInstruments) and through a signal box (PowerLab 4/20, ADInstruments) registered on a computer (software: Chart v5.0, ADInstruments). Once the catheter was in place, the pressure was left to stabilize over a period of approximately 5 to 10 minutes. The rats were sacrificed by decapitation. The hematocrit was measured post mortem by centrifuging the blood (Micro-Hematocrit Centrifuge, model MB, 11,700 RPM, 13,700 × g, International Equipment Company, MA, USA) for 10 minutes.

In the PTB experiment, invasive pressure measurements were performed as previously described [17].

### Assessment of right ventricular hypertrophy

Immediately after the rat was decapitated, the heart was removed and placed in diethylpyrocarbonate (DEPC) treated water. For gene expression analysis a piece offour times four mm of the free wall of the right ventricle was cut out, and placed in RNA*later* (Ambion Inc., TX, U.S.A.). The atria of the heart were removed and the right ventricle separated from the left ventricle and septum. Right ventricle (including the piece cut out for gene expression analysis) and left ventricle was weighed separately. Right ventricular weight to body weight ratio (RV/BW) and the entire heart weight to body weight (HW/BW) were calculated.

### Morphometric measurements

Tissue from the right ventricle was stained with reticulin that is useful to outline the architecture. The myocardial cell was considered to represent a near cylinder with a nucleus placed in the centre of the cell. Measurements were restricted to the nuclear areas. The smallest diameter was chosen as the one representing the actual diameter. The measurements were done on as many cells as possible in a field of vision. Only cells represented by a nucleus were measured. The fields of vision were randomly picked.

### Gene chip analysis

The gene expression profiles was obtained by the use of Affymetrix GeneChips (GeneChip® Rat Genome 230 2.0 Array, Affymetrix Inc., CA, U.S.A.). Protocols for the analysis of Affymetrix GeneChips and the evaluation of the sensitivity and quantitative aspects of the method have been previously described [18]. The raw data files are available in European Molecular Biology Laboratory, European Bioinformatic Institute, Microarray Informatics, (http://www.ebi.ac.uk/miamexpress). 293 genes were chosen for analysis in the hypoxic and PTB experiment (see details in the statistical analysis section).

### qPCR analysis

In the present project we used an Applied Biosystems 7000 Real-Time PCR System (Applied Biosystems, CA, U.S.A.) and probes composed of LNA™ molecules (Exiqon, Denmark). LNA (Locked Nucleic Acid) is a bicyclic nucleic acid where a ribonucleoside is linked between the 2′-oxygen and the 4′-carbon atoms with a methylene unit (CH_2_). As with the DNA probes each of the 90 LNA probes in the ProbeLibrary™ kit is labeled with fluorescein (*reporter*) and a non-fluorescent *quencher*.

Primers (forward and reverse) and probes used were: adrenergic receptor, alpha 1B (α_1B_-AR): 5′-cgtatccttgggtgccagt-3′, 5′-cacggccggtaggtgtaa-3′, probe #22; aquaporin 7: 5′-tccccggttcttc-actttc-3′, 5′-acccaccaccagttgttcc-3′, probe #15; triadin 1: 5′-gtggactacaaaaacttttcagca-3′, 5′-cagcatcgttcactagttttagagg-3′, probe #20; CD151 antigen: 5′-acggaacctgttacgcttgt-3′, 5′-cagca-atgatctccagaagga-3′, probe #5; tissue transglutaminase (tTG): 5′-agctggagagcaacaagagc-3′, 5′-gcctggtcatccaggactc-3′, probe #9; transforming growth factor, beta 1 (TGF-β1): 5′-cctgcc-cctacatttgga-3′, 5′-tggttgtagagggcaaggac-3′, probe #73; Ubiquitin C: 5′-caggacaaggagggcatc-3′, 5′-gccatcttccagctgctt-3′, probe #90; myosin regulatory light chain: 5′-cttcgcttgcttcgatgag-3′,5′-gtgagcagctccctcaggt-3′ , probe #31; clathrin, light polypeptide (Lcb): 5′-tggagagaggagc-agaagaaa-3′, 5′-cactcctgttcggtcacctt-3′, probe #89; Similar to C11orf17 protein: 5′-ctcagactc-ggggcacag-3′, 5′-atgcttcctggaccaacaga-3′, probe #40.

### Immunoblotting

Frozen samples of the right ventricle from chronic hypoxic and normoxic rats were homogenized, extracted in 300 µl lysis buffer (20 mM Tris-HCl, 5 mM EGTA, 150 mM NaCl, 20 mM glycerolphosphate, 10 mM NaF, 1% Triton X-100, 0.1% tween-20, 0.02 mM ortho-vanadate, 40 nM PMSF, PIM) and centrifuged at 3000 rpm at 4°C for 15 minutes. The supernatant was removed and protein concentration was measured by double determination of the samples against a standard curve with known concentration of albumin using Bio-Rad Protein Assay (Bio-Rad Laboratories, CA, USA). Protein lysate was mixed with sample buffer (350 mM Tris-HCl, 10% dithiothreitolsodium-laurylsulfate, 30% glycerol, 0.123% bromphenol blue). Samples, containing equal amounts of protein, and Protein Stain marker (Bio-Rad Laboratories) were loaded to the gel (Criterion XT Bis-Tris gel 4–12%, Bio-Rad Laboratories) and separation was carried out in the Criterion Electrophoresis System (Bio-Rad Laboratories) using XT-MOPS as a running buffer. The proteins were transferred from the gel to a polyvinylidine difluoride membrane for 1 hour at 100 V. The membrane was blocked in 5% milk for 2 hours at 20°C and afterwards incubated with primary antibody diluted in 5% milk over night at 4°C. The membrane was washed four times in TBS-T (stock: 10 mM tris/base, 2M NaCl, 1 mM EDTA 0.1% tween-20–200 ml stock, 1800 ml MQ-water, pH adjusted to 7.5) and then incubated in secondary antibody for 2 hours at 20°C. Then again washed four times in TBS-T and finally the blots were detected by enhanced chemiluminiscence system (ECL Plus Western Blotting Detection System, GE Healthcare UK Ltd., UK). Differences in protein abundance were determined by densitometry (Image Quant TL; Amersham Biosciences, UK). The known amount of loaded protein was used as loading control.

### Antibodies

Primary antibodies used were: anti-acetyl-Coenzyme A acyltransferase 2 (ACAA2) (cat.nr. 11111-1-AP, Ptg.Lab, IL, USA), anti-hydroxyacyl-Coenzyme A dehydrogenase/3-ketoacyl-Coenzyme A thiolase/enoyl-Coenzyme A hydratase (trifunctional protein), alpha subunit (HADHA) (cat.nr. 10758-1-AP, Ptg.Lab, IL, USA), anti-aquaporin 7 (cat.nr. AB3076, Chemicon International, MA, USA), anti-monoamine oxidase A (MAOA) (cat.nr. 10359-1-AP, Ptg.Lab, IL, USA), anti-tissue transglutaminase (tTG) (TGase II Ab-2 (TG-100), Thermo Scientific, CA, USA), anti-endothelin receptor B (ET_B_) (cat.nr. ab1921, Abcam, UK).

Secondary antibodies used were: horse radish peroxidase (HRP) conjugated goat anti-rabbit IgG (Santa-Cruz biotechnology, CA, USA), HRP conjugated goat anti-mouse IgG (Zymed, CA, USA), HRP conjugated rabbit anti-chicken (ab6753, Abcam).

### Immunohistochemistry

Pieces from the right ventricle from normoxic and hypoxic rats were stored in formalin 4% until they were embedded into paraffin. Slices of approximately 3 µm were made from each piece. Removing paraffin from the slices was done by washing in different solutions each for 5 minutes following the schedule: 2 x xylene (Xylene – mixture of isomers (AppliChem, Germany)), 2×99% ethanol, 96% ethanol, 50% ethanol and water. The slices were incubated in 3% H_2_O_2_ for 10 minutes and afterwards washed 2×5 minutes in Coon's buffer (Na_2_H_2_PO_4_ (2H_2_O), NaH_2_PO_4_ (H_2_O), NaCl – ad. 10,000 ml H_2_O). Slices were then pretreated with either citrat buffer (10 mM (Tri-sodiumcitrate dihydrat 5 mM, dinatriumhydrogencitrat 5 mM, pH adjusted to 6.0)) or TEG buffer (Tris 10 mM, EGTA 0.5 mM, pH adjusted to 9.0) for 2×5 minutes in microwave at 650 W and afterwards washed in Coon's buffer. 10% bovine serum in solution of 1% bovine serum albumin dissolved in Coon's Buffer (BSA1%) was added to avoid unspecific binding of the antibodies. The serum was removed and the primary antibody, the same as used for immunoblotting, diluted in BSA1% was added and incubating over night at 4°C in a moisture chamber.

The slices were washed in Coon's buffer and then incubating with secondary antibody (Bionylated Link - Universal LSAB™2 Kit/HRP, Rabbit/Mouse, Dako, CA, USA) for 20 minutes at 20°C in a moisture chamber. Again washing with Coon's buffer and incubation with streptavidin (Streptavidin, Dako) for 20 minutes at 20°C in a moisture chamber. After washing with Coon's buffer DAB (DAB Chromogen (tablets dissolved in Coon's buffer), Dako) was added and incubating for 5 minutes. Slices were incubated with Mayer's hematoxylin for 1 minute, washed in H_2_O and incubating for 5 minutes in each solution: 50% ethanol, 96% ethanol and 99% ethanol. Negative controls were made exactly indentical but without incubation with primary antibody.

### Statistical analysis

The data obtained in the hypoxic experiment was firstly filtered to include probe sets with at least one present call in all 28 chips and secondly logarithmic transformed. A t-test for difference between the hypoxic and the normoxic groups, that adds the effects from the four time points (allowing for different general levels at the four time points) was calculated. The corresponding P-value from the appropiate t-distribution was calculated for each gene.

The calculated P-values were used to rank the genes within each test. To estimate how many of the called genes that were falsely positively called, we estimated a false discovery rate (FDR), avoiding the assumptions of the data being independent and normally distributed. The FDR was calculated by comparing the original data to a reference distribution based on permutated data. Permutation consists in permutating group labels while keeping the size of the two groups. In this experiment we generated alternative arrangements by exchanging two individuals between the control and the intervention group for each of the four time points. For the whole experiment, there were a total of 104.976 different permutations. 500 randomly picked permutations were chosen, and for each of the 500 permutated experiments a t_sum perm._ test was calculated, and the corresponding P-values were found. FDR was found by calculating the ratio between the median number of genes based on the permutated datasets with P<0.0001 and the number of genes based on the original dataset with P<0.0001.

In the PTB experiment, the genes that were found to be significantly differentially expressed in the hypoxic experiment, were selected in the gene expression dataset belonging to the PTB experiment, and differences between the two groups were analysed using Student's t test.

Results from immunoblotting, qPCR, morphometric and hemodynamic measurements from both the PTB and hypoxic experiment were analyzed separately by using two-way analysis of variance (ANOVA) comparing hypoxia or PTB to time. In case of significance a Bonferroni post hoc test was made. Statistical calculations were made by using GraphPad Prism version 4.03 (GraphPad Software Inc, San Diego, CA, USA).

All over, P-values of less than 0.05 were considered significant.

## Results

### Functional Data

Hematocrit, SSBP, BW and HW/BW are listed in Table 2. The hematocrit was 39% in the normoxic group. Hypoxia increased the hematocrit to 50–54%. SSBP was unaltered by hypoxia. The normoxic animals gained weight during the four weeks, while the hypoxic rats had a significant lower body weight after four weeks of experiment. HW/BW in the hypoxic group showed a significant increase at week two, three and four.

**Table 2:** Parameters describing the pulmonary and systemic impact of hypoxia.

	Week 1	Week 2	Week 3	Week 4
Hematocrit (%)				
Normoxia	38.7±0.9	39.0±2.6	39.0±1.5	39.0±1.4
Hypoxia	50.2±1.3*	54.3±1.7*	51.5±1.9*	51.8±1.5*
SSBP (mmHg)				
Normoxia	131.3±2.2	127.8±2.4	124.7±4.9	124.7±2.3
Hypoxia	128.7±2.8	124.3±2.8	129.5±2.5	122.2±3.6
Body weight (gram)				
Normoxia	331±12	342±11	334±12	364±10
Hypoxia	298±11	300±11	342±10	310±19*
HW/BW (%)				
Normoxia	0.27±0.005	0.26±0.009	0.25±0.009	0.24±0.007
Hypoxia	0.29±0.004	0.31±0.008*	0.33±0.014*	0.32±0.010*

SSBP: Systemic systolic blood pressure; HW/BW: Heart weight to body weight ratio.

n = 6 in both groups at all time points. Values are means ± SE. *P<0.05 vs. normoxia at same time point.

**Figure 1:**
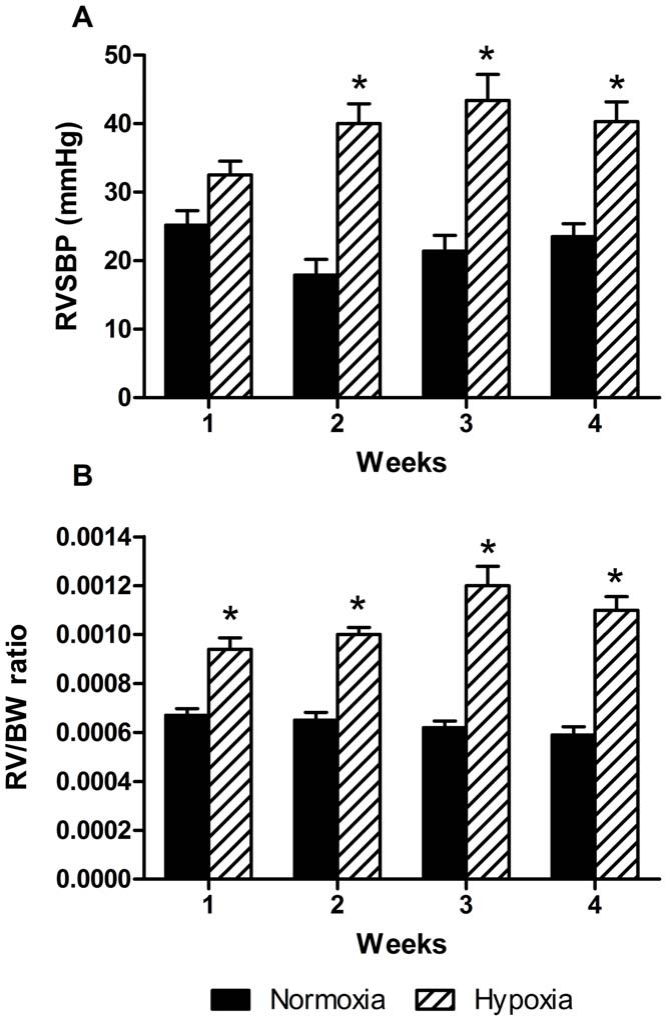
Right ventricular systolic blood pressure (RVSBP) and right ventricular hypertrophy in hypoxic experiment. A: changes in RVSBP in the normoxic and hypoxic groups at the four time points. RVSBP was constant in the normoxic group and significantly increased in the hypoxic groups after 2 weeks. B: temporal change in right ventricular hypertrophy in the hypoxic experiment assessed by right ventricular weight relative to body weight ratio (RV/BW) shows significant increase due to hypoxia. n = 6 in both groups at all time points. Values are means ± SE. *P<0.05 vs. normoxia at same time point.

RVSBP was constant in the normoxic group (18–25 mmHg) but raised by hypoxia from 32 mmHg at week one to 40–43 mmHg at week two, three and four (Figure 1A). RV/BW was almost unaltered in the normoxic group, while in the hypoxic group RV/BW was increasing during the four weeks (Figure 1B). There was a strong positive correlation between RVSBP and RV/BW in the hypoxic experiment (n = 48, R^2^ = 0.66, P<0.0001).

**Figure 2:**
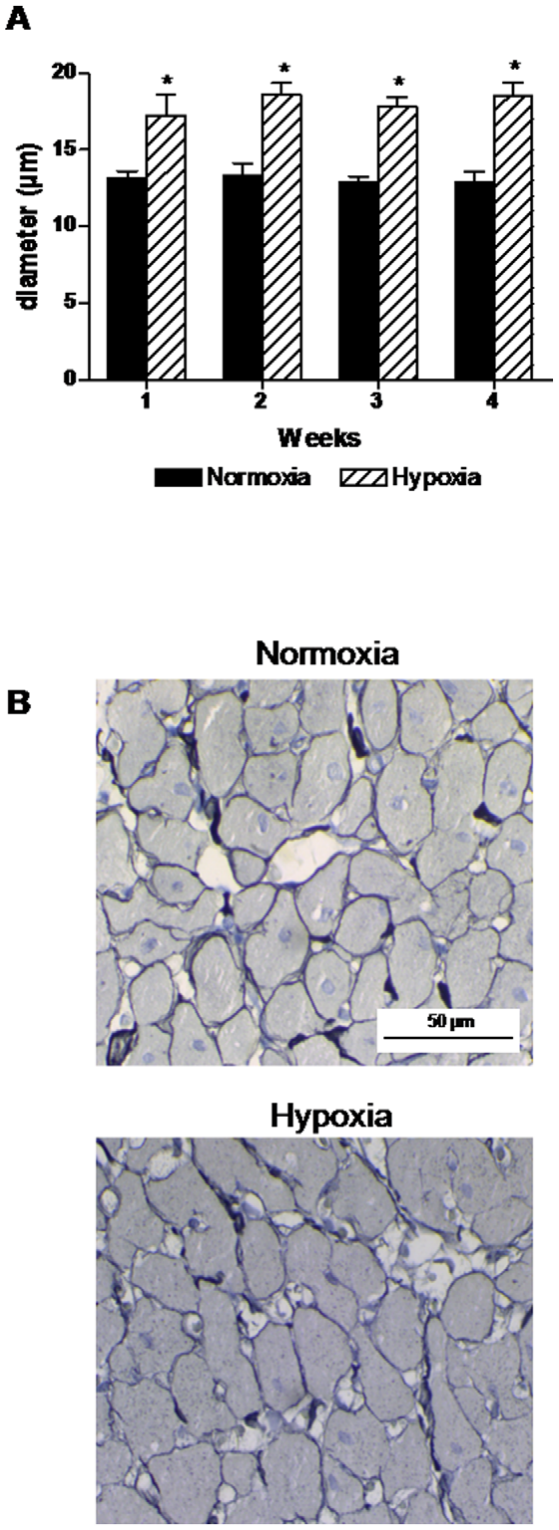
Morphometric measurements of the right ventricle. A: cardiomyocyte diameter was significantly increased by hypoxia compared to normoxic controls. B: examples of staining with reticulin in right ventricle samples from normoxic and hypoxic rats. n = 5–6 in both groups at all time points. Values are means ± SE. *P<0.05 vs. normoxia at same time point.

The diameter of the cardiomyocytes increased significantly (31 to 64%) in the hypoxic group but within each group there was no significant difference between the four different time points (Figure 2A). Staining with reticulin did not reveal any other differences between hypoxic and normoxic rats (Figure 2B).

**Figure 3:**
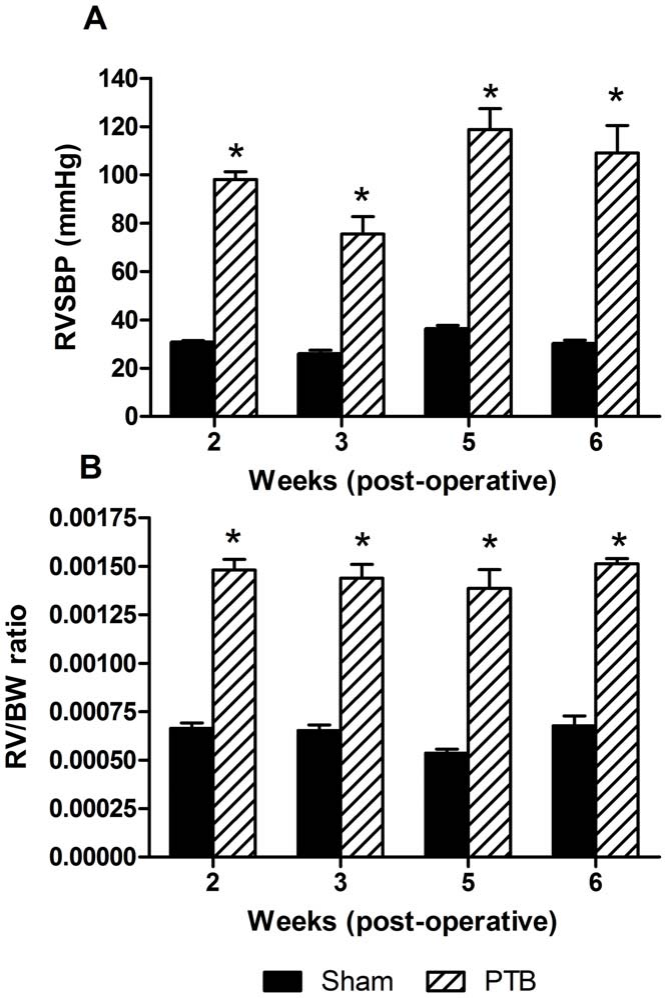
RVSBP and RV/BW in the pulmonary trunk banding (PTB) experiment. A: RVSBP was significantly increased due to PTB compared to sham operated animals. B: temporal change in right ventricular hypertrophy in the PTB experiment assessed by RV/BW and compared to the sham group shows a significant increasing effect of PTB. n = 4–7 in both groups at all time points. Values are means ± SE. *P<0.05 vs. sham at same time point.

In the PTB experiment, RVSBP was approximately constant in the sham group (26 to 38 mmHg). RVSBP was significantly raised in the PTB group at all time points (76 to 118 mmHg) (Figure 3A). Temporal change in right ventricular hypertrophy was assessed by RV/BW ratio. The degree of right ventricular hypertrophy in the PTB group was increased significantly at all time points compared to the sham group (Figure 3B). There was a strong correlation between RVSBP and RV/BW (n = 46, R^2^ = 0.76, P<0.0001).

### Gene expression data

Table 3 contains results for 49 genes from gene chip analysis for genes up- or down-regulated with a fold change greater than two.

**Table 3:** Changes in gene expression by gene chip analysis – hypoxia compared to normoxia.

Public ID	Encoded protein	Fold changes^a^
NM_012612	natriuretic peptide precursor type A	+50.13
NM_019296	cell division cycle 2 homolog A (S. pombe)	+6.21
NM_031131	transforming growth factor, beta 2	+5.60
NM_012843	Epithelial membrane protein 1	+5.25
AI145313	thymus cell antigen 1, theta	+4.64
AB026903	decay-accelerating factor	+2.31
D00688	monoamine oxidase A	+3.44
BI298356	four and a half LIM domains 1	+3.07
M14400	creatine kinase, brain	+2.95
AF413212	guanine nucleotide binding protein, alpha o	+2.91
NM_019212	actin alpha 1	+2.85
NM_012886	tissue inhibitor of metalloproteinase 3	+2.72
NM_031545	natriuretic peptide precursor type B	+2.68
L09752	cyclin D2	+2.52
AA851939	FXYD domain-containing ion transport regulator 6	+2.50
U54791	Chemokine receptor (LCR1)	+2.47
AA945643	Chitinase 3-like 1 (cartilage glycoprotein-39)	+2.38
AF095585	enigma (LIM domain protein)	+2.38
Z78279	collagen, type 1, alpha 1	+2.37
AI227742	Bcl-2-related ovarian killer protein	+2.29
NM_019237	procollagen C-proteinase enhancer protein	+2.25
BM389019	fibrillin-1	+2.23
X57764	endothelin receptor B	+2.23
AW143798	cyclin D1	+2.21
AF072892	chondroitin sulphate proteoglycan 2 (versican)	+2.16
NM_019341	Regulator of G-protein signaling 5	+2.16
NM_134452	collagen, type V, alpha 1	+2.11
AB026665	peptide/histidine transporter PHT2	+2.11
NM_019386	tissue transglutaminase	+2.08
AW253722	RAB13	+2.01
AI009597	FXYD domain-containing ion transport regulator 3	−8.67
NM_031543	cytochrome P450, subfamily 2E, polypeptide 1	−5.60
AF385402	Potassium channel, subfamily K, member 2	−4.15
AI535411	myosin heavy chain, polypeptide 7	−3.63
AJ243304	triadin 1	−3.04
NM_019157	Aquaporin 7	−2.82
BE103537	tropomyosin 1, alpha	−2.81
U31554	limbic system-associated membrane protein	−2.76
AI137995	sodium channel, voltage-gated, type IV, beta	−2.50
NM_012505	ATPase, Na+K+ transporting, alpha 2	−2.37
NM_080399	Smhs1 protein	−2.18
NM_057197	2,4-dienoyl CoA reductase 1, mitochondrial	−2.14
NM_030865	Myocilin	−2.14
AF322216	immunoglobulin superfamily, member 1	−2.14
NM_017079	CD1d1 antigen	−2.13
NM_012793	guanidinoacetate methyltransferase	−2.08
AA899304	acetyl-coenzyme A acetyltransferase 1	−2.05
AW528891	potassium voltage gated channel, Shal-related family, m…	−2.03
NM_016991	adrenergic receptor, alpha 1B	−2.03

a Values are the average of a four-way analysis as described in Methods. Only genes with fold changes larger than 2 are included in this table.

**Figure 4:**
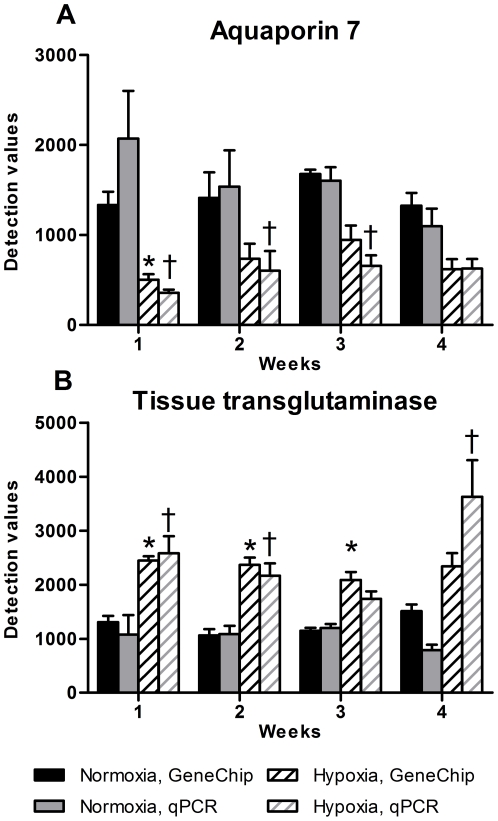
qPCR analysis of aquaporin7 and tissue transglutaminase. A: gene expression of the aquaporin 7 mRNA by use of gene chip and qPCR shows a decrease in gene expression according to hypoxia. B: gene expression of tissue transglutaminase mRNA by use of gene chip and qPCR shows the opposite impact of hypoxia by increasing gene expression. n = 6 in both groups at all time points.

**Figure 5:**
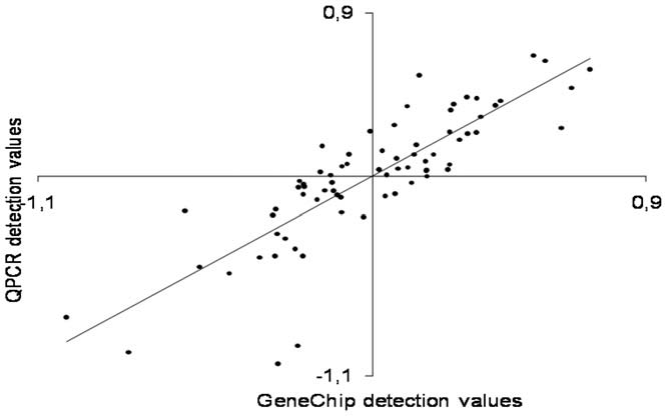
Correlation of the gene chip and qPCR gene expression results. The correlation is tested by comparing three upregulated, four downregulated and two not regulated genes (according to the gene chip, tsum analysis). There was found significant correlation between qPCR and gene chip analysis. Values are means ± SE. *P<0.05 vs. normoxia, gene chip at same time point, †P<0.05 vs. normoxia, qPCR at same time point.

The validation of the gene expression profiles was done by qPCR. We chose four upregulated (CD 151 antigen, tTG, TGF-β1 and Similar to C11orf17 protein), three downregulated (α_1B_-AR, aquaporin 7, and triadin 1) and two genes without change (myosin regulatory light chain and clathrin – light polypeptide). Ubiquitin C was used for normalization. The qPCR results were in accordance with the gene chip data (Figure 4A,B), exemplified by aquaporin 7 and tTG. The correlation between the gene chip and qPCR results was tested by comparing 3 up-regulated, 4 down-regulated and 2 genes with no change in expression according to the gene chip tsum analysis. There is a strong positive correlation between gene chip detection (normalized to 0) values and the qPCR detection values (normalized to 0) and in the hypoxic experiment (n = 72, R^2^ = 0.70) (Figure 5).

**Figure 6:**
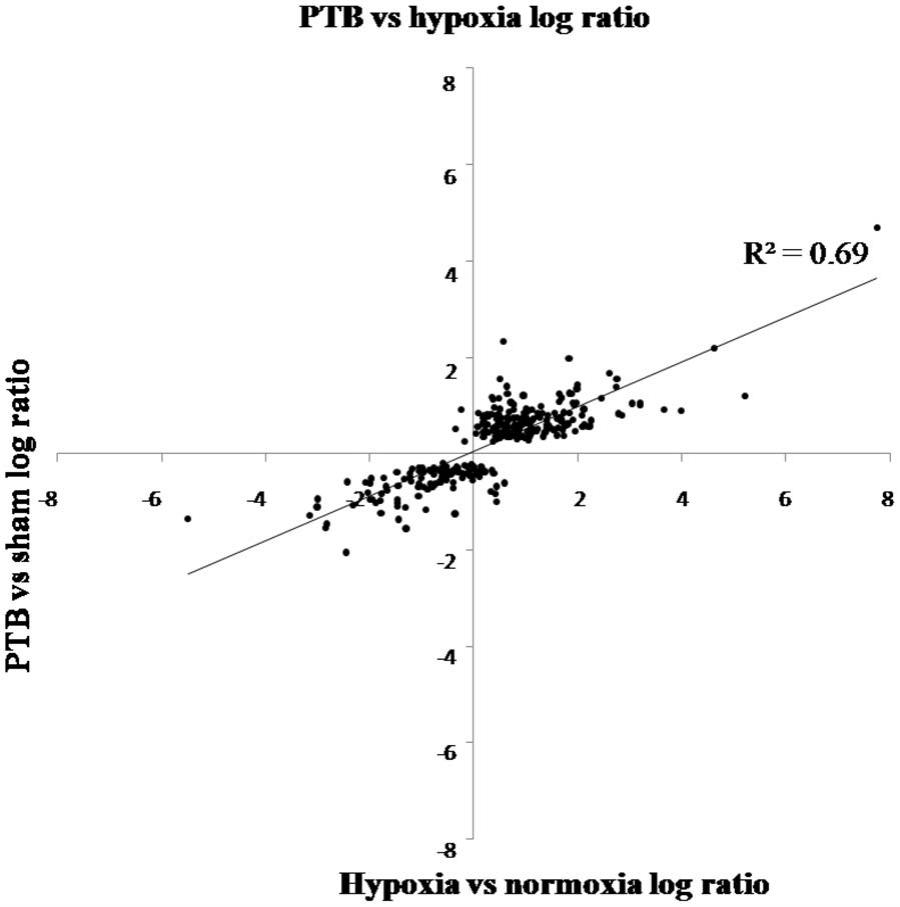
Log ratio of gene chip data obtained in the right ventricle of chronic hypoxic compared to normoxic rats versus pulmonary trunk banded (PTB) compared to sham-operated rats. There was found a positive correlation between regulation of the genes in the two experiments with a significant correlation coefficient R^2^ = 0.69 (P<0.05, n = 288).

The signal log ratio estimates the magnitude and direction of change of a transcript when two arrays are compared (experiment versus control). Signal log ratios from the hypoxic and the PTB experiments were plotted for 288 genes (5 genes were excluded of the original 293 genes tested because they showed absent calls in the PTB experiment). 266 were regulated in the same direction or were unchanged in the two experiments (Wilcoxon signed rank test: P<2.2×10^−16^). Of these genes, 172 were non-expressed sequence tags (non-EST) involved in apoptosis, inflammation, heart function, and growth and the remaining 94 were EST. Of the 22 genes regulated differently, 11 were up-regulated and 5 unaltered in the PTB experiment but down-regulated in the hypoxic experiment, 3 were down-regulated in PTB and up-regulated in hypoxia and 3 were EST (Table 4). There is a strong positive correlation between the log ratios of gene expression in the hypoxic and the PTB experiment (R^2^ = 0.69, P<0.05, n = 288) (Figure 6). Limiting the analysis to genes where the fold change is larger than 2 yielded an even stronger correlation (R^2^ = 0.84, P<0.05, n = 125) of gene expression in the hypoxic and PTB experiment.

**Table 4:** Differentially regulated genes in the right ventricle of pulmonary trunk banded (PTB) rats versus chronic hypoxic rats.

Public ID	Encoded protein	Fold change	
		PTB	Hypoxia
NM_024400	A disintegrin and metalloproteinase with thrombospondin motifs 1 (ADAMTS-1)	0.60	−0.61
BG671506	avian sarcoma virus CT10 (v-crk) oncogene homolog	0.38	−0.41
AI454911	endothelial monocyte activating polypeptide 2	0.15	−0.28
BF282186	eukaryotic initiation factor 5 (eIF-5)	0.35	−0.35
AI713966	insulin-like growth factor binding protein 3	0.41	−0.84
NM_012588	insulin-like growth factor binding protein 3	0.33	−0.78
NM_053713	Kruppel-like factor 4 (gut)	0.48	−0.68
AA818911	mob protein	0.21	−0.39
AI409862	protein phosphatase 1, catalytic subunit, beta isoform	0.22	−0.37
NM_033096	protein phosphatase 1B, magnesium dependent, beta isoform	0.12	−0.36
AY043246	regulator of G-protein signaling protein 2	0.44	−0.99
AI454052	c-kit receptor tyrosine kinase	−0.24	0.91
AW914928	DEAD (aspartate-glutamate-alanine-aspartate) box polypeptide 25	−0.36	0.51
BI275583	lectin, galactoside-binding, soluble, 2 (galectin 2)	−0.17	0.15

In addition 4 genes (LYRIC, glioma tumor suppressor candidate region gene 2, splicing factor (arginine/serine-rich 5), transducer of ERBB2 1, and TRAP-complex gamma subunit) were unaltered in PTB, but down-regulated in hypoxia, but with fold changes less than 0.5.

### Immunoblotting

**Figure 7:**
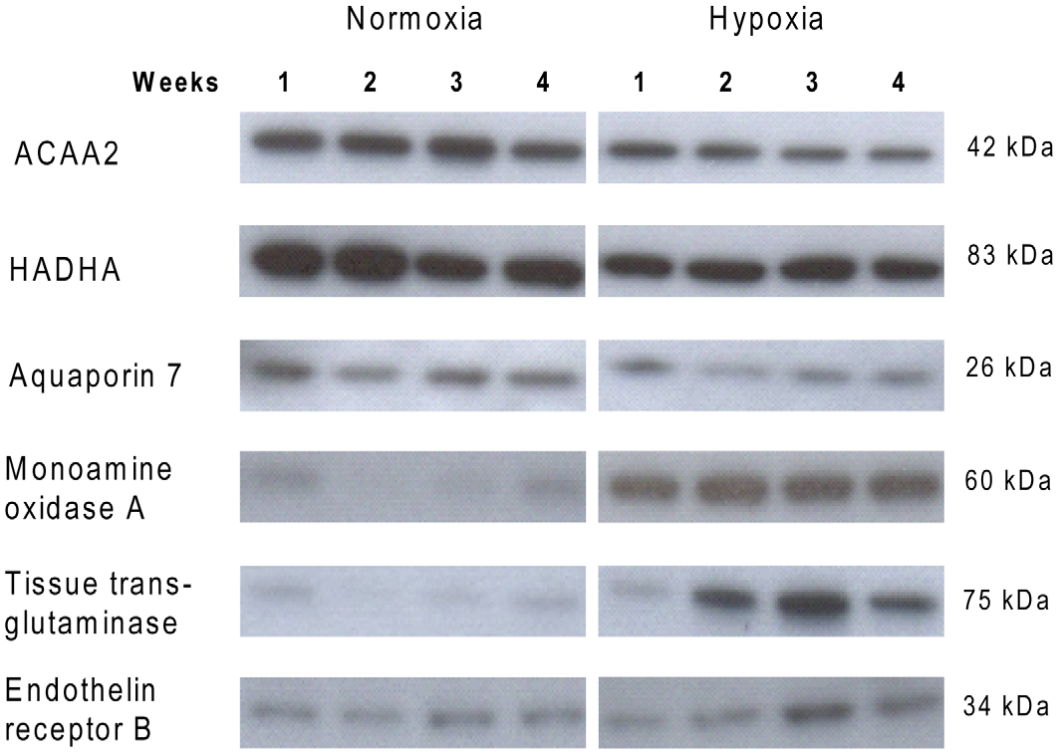
Representative immunoblots for proteins measured in the right ventricle from normoxic and hypoxic rats. ACAA2: acetyl-Coenzyme A acyltransferase 2, HADHA: hydroxyacyl-Coenzyme A dehydrogenase/3-ketoacyl-Coenzyme A thiolase/enoyl-Coenzyme A hydratase (trifunctional protein), alpha subunit.

**Figure 8:**
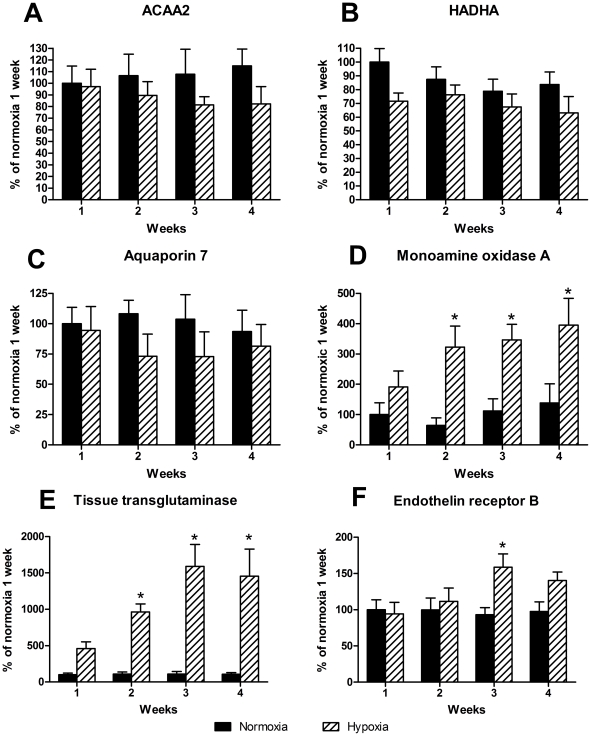
Immunoblottings of right ventricle samples from normoxic and hypoxic rats. Proteins participating in metabolism A: ACAA2, B: HADHA and C: aquaporin 7 show tendency to downregulation by hypoxia. D: monoamine oxidase A, E: tissue transglutaminase and F: endothelin receptor B were all upregulated by hypoxia. ACAA2: acetyl-Coenzyme A acyltransferase 2, HADHA: hydroxyacyl-Coenzyme A dehydrogenase/3-ketoacyl-Coenzyme A thiolase/enoyl-Coenzyme A hydratase (trifunctional protein), alpha subunit. Values are means ± SE and are calculated as percent of normoxia 1 week. n = 6 in both groups at all time points. *P<0.05 vs. normoxia at same time point.

Figure 7 shows the representative immunoblots. Six proteins were chosen for immunoblotting based on the gene chip that showed three genes were downregulated (ACAA2, HADHA and aquaporin 7) and three were upregulated (tTG, MAOA and ET_B_). By use of antibodies against these proteins we showed that some of the changes found on gene levels are also reflected at protein level. ACAA2, HADHA and aquaporin 7 were not significantly downregulated, but the tendency is clear (Figure 8A–C). MAOA and tTG were significantly upregulated in hypoxic rats after 2, 3 or 4 weeks when compared to normoxic controls (Figure 8D–E). Finally, ET_B_ was only significantly upregulated after 3 weeks of hypoxia (Figure 8F).

### Immunohistochemistry

**Figure 9:**
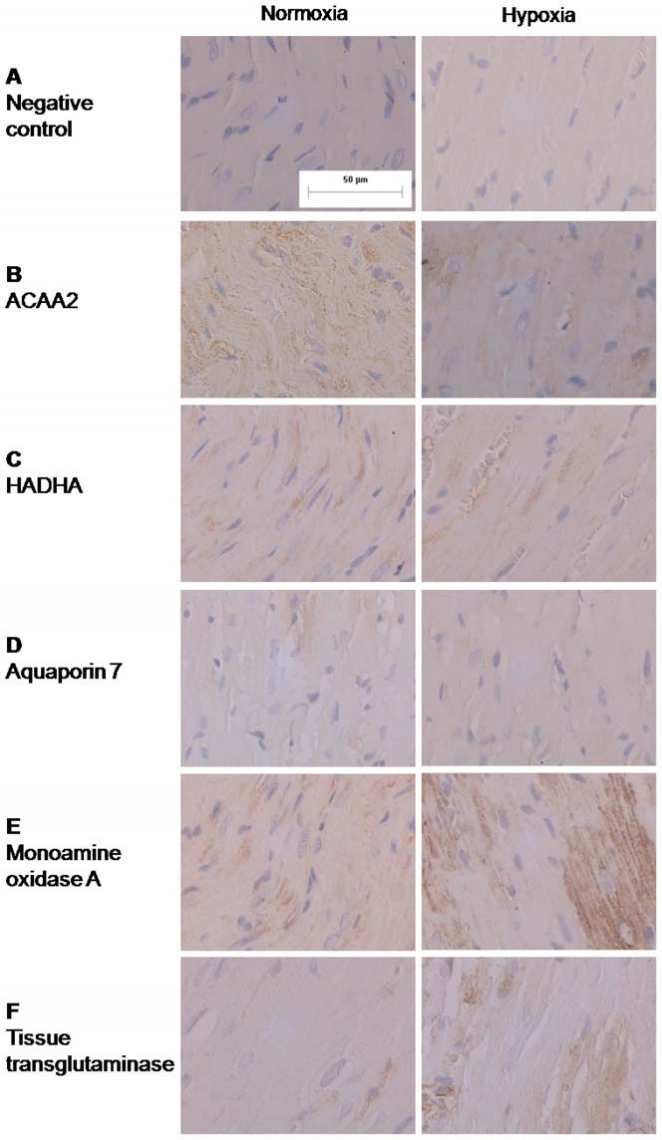
Immunostainings of slides from the right ventricle from rats exposed to normoxia or hypoxia for 2 weeks. A: Negative controls without incubation with the primary antibody. The bar on control picture shows the size reference (50 µm) for all pictures. B: ACAA2 C: HADHA, D: aquaporin 7, E: monoamine oxidase A, F: tissue transglutaminase. All proteins were found to be located to the cardiomyocyte and only aquaporin 7 was thought to be located to the cellular membrane whereas the other proteins is located to the cytosol.

By incubating slides from the right ventricle from hypoxic and normoxic rats with antibodies used for immunoblotting we were able to evaluate the localization of the proteins. Controls were made without primary antibody incubation (Figure 9A). For the proteins participating in beta-oxidation, ACAA2 and HADHA, the localization is intracellular in the cardiomyocyte (Figure 9B–C). Aquaporin 7 was also located in the cardiomyocyte at the cellular membrane (Figure 9D). MAOA was also upregulated on the immunostainings when compared to normoxic controls and the location is intracellular (Figure 9E). Finally, tTG was also located to the cardiomyocyte mainly in the cytosol (Figure 9F).

## Discussion

The main findings of the present study are addressing gene expression common for the pressure loading of the right ventricle in both chronic hypoxic rats and rats with banding of the pulmonary trunk. The present study revealed alterations in expression of 172 genes involved in apoptosis, inflammation, heart function, and growth. A small subset of differentiated genes in the hypoxia and PTB groups suggests pressure load as the main contributer to development of right ventricular hypertrophy. GeneChip analysis of the right ventricle was confirmed by qPCR for a subgroup of genes and was further substantiated by measuring protein expression showing a marked upregulation of tTG due to right ventricular hypertrophy.

### Role of hypoxia versus pressure load in right ventricular hypertrophy in pulmonary hypertension

The hypobaric hypoxia model has been used in several studies for induction of pulmonary hypertension in rats and has shown increase of RVSBP and right ventricular weight [19]. Another approach to induce high RVSBP and to increase right ventricular weight is by PTB [17], [20]. By comparing these two animal models, it is possible to distinguish the isolated effect of respectively pressure load and hypoxia. There was a significant correlation of genes changed in the right ventricle of chronic hypoxic rats with the changes observed in the PTB experiment and this correlation was even stronger when only genes regulated more than two-fold were considered. These findings suggest that pressure load is the main factor altering gene expression in the right ventricle in rats with pulmonary hypertension induced by hypoxia.

Comparing chronic hypoxia and PTB identified a small subgroup of genes changing in opposite directions suggesting that hypoxia alone has an impact on the gene expression in the right ventricle. Thus, c-kit receptor tyrosine kinase and DEAD (asparatate-glutamate-alanine-asparatate) box polypeptide 25 were upregulated in hypoxia and downregulated in the PTB experiments, while insulin-like growth factor binding protein 3 and regulator of G-protein signaling protein 2 were upregulated. Both c-kit receptor tyrosine kinase and insulin-like growth factor binding protein 3 have previously been described to be involved in hypoxia-induced angiogenesis [21], [22], and insulin growth factor 1 stimulation in mouse fibroblasts resulted in upregulation of DEAD [23]. Therefore, hypoxia seems to directly affect gene expression in the right ventricle. However, in all cases the fold changes in gene expression comparing the PTB and hypoxic experiments with regard to differentially expressed genes are small (Table 4), and it cannot be excluded that differences in pressure load comparing chronic hypoxic rats and PTB rats may also contribute to the differential gene regulation.

Previous studies have also provided evidence suggesting that mechanical load of the right ventricle from rats with pulmonary hypertension influences gene expression [11]. Thus, atrial natriuretic peptide expression, probably induced by stretch of the myocardium, was upregulated in the right ventricle from rats with pulmonary hypertension induced by either moncrotaline or hypoxia [8], [9], and in agreement with these findings, both natriuretic peptide precursor type A and B were markedly increased in the present study. Genes involved in cell proliferation, the cyclin family of genes and BCl2, were upregulated in the right ventricle of rats with pulmonary hypertension induced by monocrotaline [9], [10], and the same was the case for cyclin D1 and D2 as well as BCl2 in the present study. In addition, several signaling processes involving fetal gene re-expression, activation of protein translocation, increase in mass, and enlargement of cell size/volume have been identified as markers of hypertrophy as a response to hemodynamic overload [24]. In the present study the diameter of the cardiomyocytes was increased, and alpha-actin expression was upregulated together with four and a half LIM domains 1 (FHL), and enigma (LIM domain protein). FHL is contained in a complex within the cardiomyocyte sacromere and mice lacking FHL displayed a blunted hypertrophic response suggesting FHL1 to mediates hypertrophic biomechanical stress responses in the myocardium [25], while the Enigma protein family are Z-line proteins at the border between two sarcomers [26]. Thus, upregulation of a series of genes in the present study also suggest that mechanical load regulate gene expression and results in right ventricular hypertrophy.

### Role of specific signal pathways changed in right ventricular hypertrophy

During development of right ventricular hypertrophy the myocardium changes metabolism to avoid ischemia [27]. Normally the major substrate for heart metabolism is free fatty acids that account for 60–80%. The remaining part comes from metabolism of carbohydrates, but during development of left ventricular hypertrophy and heart failure the ratio alters towards increased carbohydrates as cardiac fuel substrate and augmented mitochondrial respiratory capacity which is considered to play a central role in hypoxia-mediated cardioprotection [27]. A study of gene expression from chronic hypoxic rats showed increased expression of genes associated to glucose metabolism and they also found changes in the left ventricle, which indicates that not only myocardial hypertrophy causes changes, but also chronic hypoxia contributes to altered gene expression [8]. Indeed, in the present study genes encoding for enzymes participating in beta-oxidation of fatty acids (ACAA2 and HADHA) were downregulated in right ventricles from hypoxic rats. The tendency was reflected at protein level, although not significantly and supports that pressure load by itself is able to cause a shift in genes related to myocardial metabolism from free fatty acids to carbohydrates.

Aquaporin 7 is a water and glycerol channel that has been found especially in adipocytes and skeletal muscle cells in the human body. The overall function of aquaporins is to maintain cellular water homeostasis [28], [29]. Studies of aquaporin 7 showed that it is expressed in cardiac tissue from mice, rats and humans [30]. Our results confirmed these findings both by gene chip, qPCR and immunoblotting. Moreover, we found that hypoxia decreases gene expression for aquaporin 7, although this was not confirmed at protein level. Skowronski et al. found that aquaporin 7 is only localized in small vessels in cardiac tissue, and these observations agree with our findings [31]. A downregulation of aquaporin 7 in hypoxic rats may reflect reduced glycerol transport as a consequence of a shift of the metabolism from fatty acids to carbohydrates.

Hypertrophy of the ventricle also leads to remodeling of the ventricular wall and altered expression of structural proteins in the myocardium and in the surrounding tissue. Studies of tTG in left the ventricle show association between the expression of tTG and development of ventricular hypertrophy [32], [33]. The mechanism is primarily through its action as TGase leading to structural changes of actin and myosin, but also more or less through the GTPase activity. tTG is coupled to the α_1B_-AR as Gα-protein [34]. Overexpression of α_1B_-AR is known to induce cardiac hypertrophy [35] and studies of the expression of the α_1B_-AR have shown that it is downregulated on mRNA level in vascular smooth muscle cells from chronic hypoxic animals [36], and that knock-out of the receptor did not alter development of right ventricular hypertrophy and the increase in RVSBP [37]. The conclusion of these studies is that α_1B_-AR is associated to vascular smooth muscle cell proliferation. Our findings show that the α_1B_-AR is downregulated in the right ventricle at mRNA level, while the potential coupling protein tTG is markedly upregulated and associated to right ventricular hypertrophy in rats with pulmonary hypertension. The exact role of α_1B_-AR is still unknown but it seems to play an adaptional role to avoid development of cardiac hypertrophy according to pulmonary hypertension.

Transforming growth factor beta 1 (TGF-β1) is thought to be associated with proliferation of cells during development of hypertrophy and cell division. Studies of rats with pulmonary hypertension and right ventricular hypertrophy induced by monocrotaline showed by qPCR analysis increased levels of TGF-β1 in the right ventricle but not in the left ventricle indicating association to right ventricular hypertrophy [38]. Also immunoblottings of pulmonary arteries from chronic hypoxic rats showed association between TGF-β1 and increased proliferation of vascular smooth muscle cells [39]. These findings indicate that TGF-β1 is associated both to right ventricular hypertrophy and vascular smooth muscle cell proliferation. Our studies support that TGF-β1 appears to play a role in development of right ventricular hypertrophy.

MAOA is an enzyme located to the mitochondria of the cardiomyocytes and metabolizes epinephrine, norepinephrine, and serotonin (5-HT). Studies have shown that 5-HT is associated to ventricular hypertrophy by binding to its receptor 5-HT_2B_, and that it induces oxidative stress and apoptosis [40]. It has been found that blocking of the 5-HT_2B_ receptor only partly inhibited the effect of 5-HT, and that inhibition of MAOA prevented the hypertrophic effect of 5-HT [41]. Overexpression of the 5-HT_2B_ receptor leads to left ventricular hypertrophy. The localization of MAOA has been found to be intracellular [42], [43]. Our findings indicate an association between right ventricular hypertrophy and the expression of MAOA. Moreover, we evaluated the localization of MAOA and found that it is located to the cardiomyocytes and probably to the mitochondria, which are highly expressed in cardiomyocytes and is the place where catecholamines and 5-HT are metabolized. Reactive oxygen species (ROS), a product from oxidation of 5-HT catalyzed by MAOA, is related to right ventricular hypertrophy and ROS has been found to be located to the mitochondria [44]. This indicates that metabolization of 5-HT and thereby MAOA is located here.

The effects of endothelin are mediated by two distinct receptors termed ET_A_ and ET_B_, where 90% of endothelin receptors belong to the ET_A_ subtype in cardiomyocytes, and their stimulation has a positive inotropic effect [45]. Cardiac ET_B_ receptors may contribute to clearance of circulating endothelin and together with the ET_A_ to cardiac fibrosis and cardiomyocyte hypertrophy [45], [46]. In the present study only the ET_B_ receptor expression was elevated in the right ventricle as well as expression of several collagens e.g. collagen type 1 alpha 1 and collagen type V alpha 1 (Table 3). The dual ET_A_/ET_B_ receptor antagonist, bosentan reduces right ventricular hypertrophy in pulmonary hypertension in chronic hypoxic rats [47], but at present it is unclear whether the block of endothelin clearance and pulmonary vascular dilation by ET_B_ receptors outweigh the beneficial effects of blocking both the ET_A_ and ET_B_ receptors in pulmonary hypertension due to hypoxia.

In conclusion, we have found that several genes are altered during development of right ventricular hypertrophy induced by pulmonary hypertension in chronic hypoxic rats. In case of the metabolic genes the effect of high pressure on the right ventricle appears compensated at the protein level, while both expression of genes and proteins of importance for myocardial function and remodelling are altered by the increased pressure load of the right ventricle. These findings imply that treatment of pulmonary hypertension, in addition to reduction of pulmonary vascular resistance, should also aim at reducing right ventricular pressure or by direct effects on the heart limit the organ damaging effects of high pulmonary pressure.
